# RNA therapeutics in the clinic

**DOI:** 10.1002/btm2.10374

**Published:** 2022-07-06

**Authors:** Alexander Curreri, Disha Sankholkar, Samir Mitragotri, Zongmin Zhao

**Affiliations:** ^1^ John A. Paulson School of Engineering and Applied Sciences Harvard University Cambridge Massachusetts USA; ^2^ Wyss Institute for Biologically Inspired Engineering at Harvard University Boston Massachusetts USA; ^3^ Lexington High School Lexington Massachusetts USA; ^4^ Department of Pharmaceutical Sciences, College of Pharmacy University of Illinois at Chicago Chicago Illinois USA; ^5^ University of Illinois Cancer Center Chicago Illinois USA

**Keywords:** antisense oligonucleotide, aptamer, clinical trial, gene therapy, lipid nanoparticle, mRNA, mRNA vaccine, RNA therapeutic, siRNA

## Abstract

Ribonucleic acid (RNA) therapeutics are being actively researched as a therapeutic modality in preclinical and clinical studies. They have become one of the most ubiquitously known and discussed therapeutics in recent years in part due to the ongoing coronavirus pandemic. Since the first approval in 1998, research on RNA therapeutics has progressed to discovering new therapeutic targets and delivery strategies to enhance their safety and efficacy. Here, we provide an overview of the current clinically relevant RNA therapeutics, mechanistic basis of their function, and strategies to improve their clinical use. We discuss the 17 approved RNA therapeutics and perform an in‐depth analysis of the 222 ongoing clinical trials, with an emphasis on their respective mechanisms and disease areas. We also provide perspectives on the challenges for clinical translation of RNA therapeutics and suggest potential strategies to address these challenges.

**List of Abbreviations**
2'‐MOE: 2'‐*O*‐methoxyethyl ribose substitutions
2'‐Ome: 2'‐*O*‐methyl ribose substitution
ADARs: adenosine deaminases acting on RNA
AGO2: argonaute 2
AGT: alanine‐glyoxylate aminotransferase
ALAS: aminolevulinic acid synthase
Anvisa: Brazilian Health Regulatory Agency
ASCVD: atherosclerotic cardiovascular disease
ASO: antisense oligonucleotide
Cas: CRISPR associated proteins
cEt: constrained ethyl bridge nucleic acid substitution
CMV: cytomegalovirus
COVID‐19: coronavirus disease 19
CRISPR: Clustered regularly interspaced short palindromic repeats
DMD: Duchenne muscular dystrophy
dsRNA: double‐stranded ribonucleic acid
EMA: European Medicines Agency
FAP: familial amyloidotic polyneuropathy
FDA: Food and Drug Administration
FH: familial hypercholesterolemia
GalNAc: *N*‐acetylgalactosamine
GO: glycolate oxidase
HC: Health Canada
HeFH: heterozygous familial hypercholesterolemia
HoFH: homozygous familial hypercholesterolemia
IM: intramuscular
IV: intravenous
IVT: intravitreal
IVTrnsc: in vitro transfection
LCA10: Leber congenital amaurosis 10
LDL: low‐density lipoprotein
LDLC: low‐density lipoprotein cholesterol
LDLR: low‐density lipoprotein receptor
LNP: lipid nanoparticle
mAbs: monoclonal antibodies
MFSD8: major facilitator superfamily domain containing 8
mRNA: messenger ribonucleic acid
NAFLD: nonalcoholic fatty liver disease
NASH: nonalcoholic steatohepatitis
PCSK9: proprotein convertase subtilisin‐kexin type 9
PEG: polyethylene glycol
PH1: primary hyperoxaluria type 1
PMDA: Japanese Pharmaceuticals and Medical Devices Agency
PMO: phosphorodiamidate morpholino oligonucleotide
PS: phosphorothioate
RISC: ribonucleic acid‐induced silencing complex
RNA: ribonucleic acid
SARS‐CoV‐2: severe acute respiratory syndrome coronavirus 2
SC: subcutaneous
siRNA: small‐interfering ribonucleic acid
SMA: spinal muscular atrophy
SMN1/SMN2: survival motor neuron 1/2
srRNA: self‐replicating ribonucleic acid
ssRNA: single‐stranded ribonucleic acid
TTR: transthyretin
UBE3A: ubiquitin‐protein ligase 3A gene
VEGF165: vascular endothelial growth factor 165
wet AMD: neovascular macular degeneration

## 1 INTRODUCTION

Ribonucleic acid (RNA) therapeutics are an emerging class of therapeutic modalities, which derive their function from the genetic material that they imitate. RNA therapeutics take on various forms and this review will largely focus on messenger RNA (mRNA), antisense oligonucleotides (ASOs), small‐interfering RNA (siRNA), and aptamers. These therapeutics have been used to treat diverse diseases by regulating protein production and function, and in doing so, various biological functions. The first clinical approval of an RNA therapeutic came in 1998 when the Food and Drug Administration (FDA) approved Vitravene (fomivirsen) for the treatment of retinitis caused by cytomegalovirus. There have since been 16 additional approvals with the most recent approval in early 2022 when Spikevax (COVID‐19 vaccine, mRNA) was upgraded from emergency use authorization. While RNA therapeutics have been studied in research laboratories for over 30 years,^1^, ^2^ the recent approvals of Spikevax and Comirnaty (tozinameran) as COVID‐19 vaccines have brought RNA therapeutics, especially mRNA therapeutics, to the forefront of medicine. In this review, we discuss the clinical translation of this rapidly growing class, RNA therapeutics. In particular, we discuss the biological basis of RNA therapeutics, their function, and key approaches to deliver RNA payloads to the target sites. Additionally, we overview the clinical landscape of 17 approved products and 222 ongoing clinical trials. We also provide a perspective on current challenges and future outlook.

## 2 BIOLOGICAL BASIS, APPLICATIONS, POTENTIAL, AND ADVANTAGES OF RNA THERAPEUTICS

**FIGURE 1:**
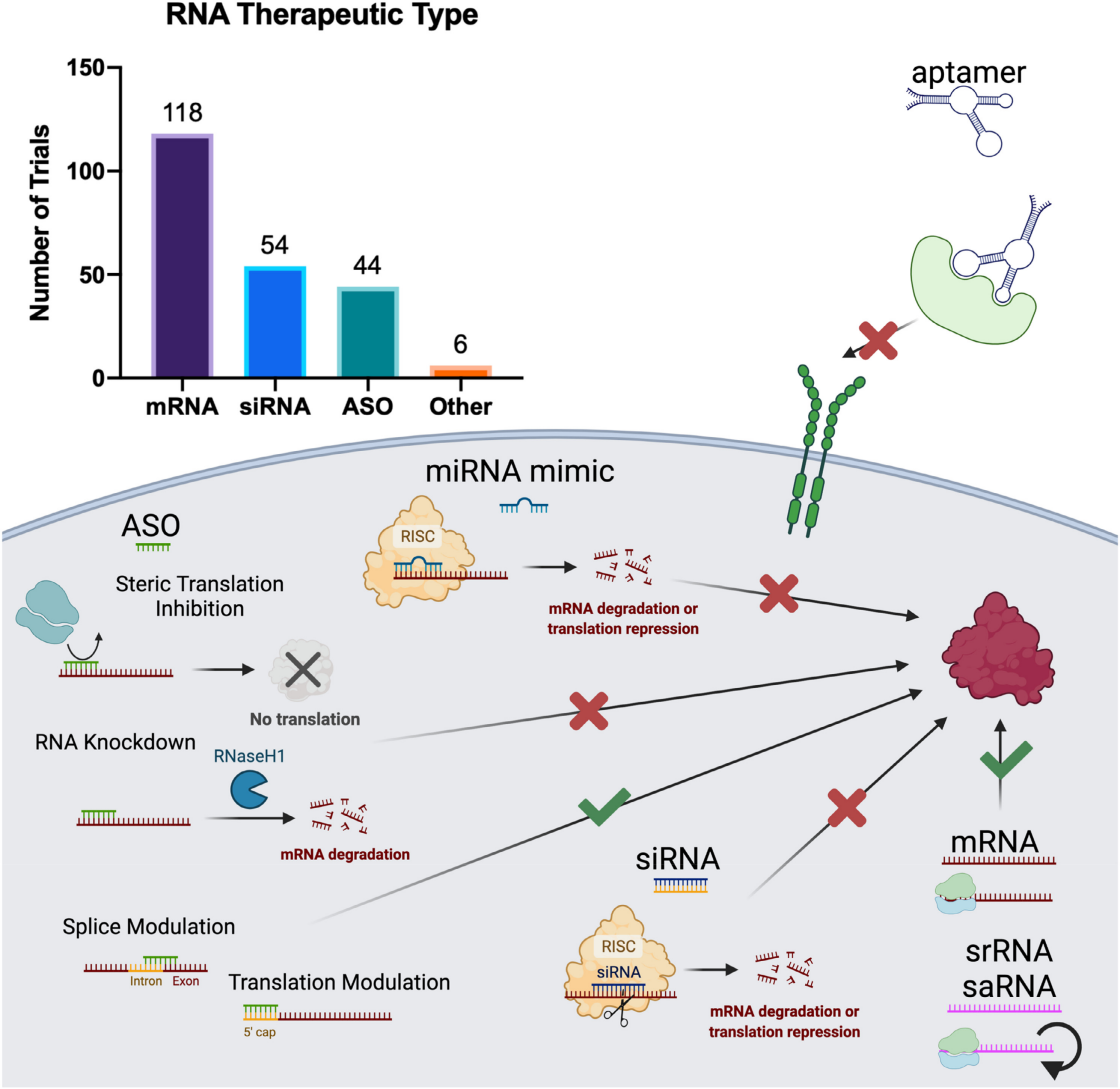
Summary of RNA therapeutic ongoing clinical trial landscape with a schematic representation of the mechanisms of action involved in different classes of RNA therapeutics. Created with BioRender.com

The central dogma of RNA therapeutics is the modulation of protein function and/or production, either by directly targeting proteins, interfering with RNAs encoding the relevant proteins, or providing the genetic code for protein production. There are three major ways in which this central goal is accomplished: (i) binding and blocking of proteins using aptamers; (ii) targeting and binding to native RNAs using ASOs, siRNA, and miRNA mimics; and (iii) expressing target proteins using mRNA.^3^ A schematic representation of the mechanisms exploited by RNA therapeutics can be found in Figure 1.

mRNAs are currently the most investigated RNA therapeutic type in active clinical studies.^4^, ^5^ mRNA in healthy cells is transcribed from DNA and then translated to proteins. Several efforts have been made to deliver exogenous mRNAs to encode particular proteins of interest (Figure 1). These exogenous mRNAs, which are single‐stranded RNA (ssRNA) fragments that range from 2000 to 20,000 base pairs, have largely been used to code for antigens for vaccination or therapeutic proteins for direct disease intervention.^6^ Significant work has been done to improve mRNA stability and translation. Major sequence modifications made to the coding strand include addition of untranslated regions and caps to the 5′ end and untranslated regions flanked by polyadenylation tails to the 3′ end.^7^ Additionally, codon optimization, to remove rare codons from the sequence, is thought to be important for enhancing translation,^8^ but it may represent a potential cause for safety concern.^9^ While optimization of stability and translation has improved the efficacy of mRNA therapeutics, a major challenge that has stymied their preclinical and clinical success is the difficulty in delivering them into the target cells. A variety of carriers have been explored to solve mRNA delivery challenges including lipid nanoparticles (LNPs),^10^, ^11^ protamine conjugates,^12^, ^13^ nanoemulsions,^14^ liposomes,^15^, ^16^ ^,^ and a variety of other nanoparticles.^17^, ^18^ These carriers stabilize and protect mRNAs, augment cellular uptake, and promote endosomal escape of the payloads, which eventually leads to protein production. Extensive reviews of mRNA delivery systems can be found in recently published reviews elsewhere.^19^, ^20^

The most successful, in terms of number of approved products, of the RNA therapeutics are ASOs. ASOs are single‐stranded oligonucleotides that target endogenous RNA, including noncoding RNA (ncRNA) and mRNA. ASOs are usually between 13 and 30 base pairs long and rely on complementarity for their function.^21^ ASOs' mechanisms of action vary depending on the target, but there are four major considerations for mRNA and pre‐mRNA therapies including (i) RNA knockdown, (ii) steric translation inhibition, (iii) splice modulation, and (iv) translation modulation (Figure 1). RNA knockdown and steric translation inhibition aim to decrease protein expression while splice modulation and translation modulation increase protein expression. For RNA knockdown, ASOs contain small segments of DNA, which are used to directly bind to the target site on target pre‐mRNA or mRNA via complementary base pairing.^22^ RNase H1 recognizes the DNA–RNA duplex and cleaves the phosphodiester bond by hydrolysis,^23^ thus destroying the pre‐mRNA or mRNA and lowering protein levels in the cell.^24^ Steric translation inhibition occurs when an ASO directly binds mRNA at a location that is close to the start codon of the relevant exon. This event sterically blocks ribosomes and transfer RNAs from associating with the relevant exon and subsequently prevents initiation of translation.^25^ Additionally, ASOs have been used to increase protein expression. Splice modulation is a mechanism by which ASOs binding to pre‐mRNA near exons blocks splice enhancers or repressors. This results in an alteration of normal splicing function in cells and can be used to skip unwanted mutations or include previously excluded exons.^26^ ASOs can also increase protein expression by translation modulation. By binding the 5′ untranslated region, upstream of the relevant exon, ASOs can decrease the ribosome attachment to that region, resulting in an increased likelihood of ribosome attachment at the start codon.^27^ There are additional mechanisms that have been explored to target endogenous translation silencing molecules like microRNA and other ncRNAs.^28^, ^29^, ^30^ A major challenge for ASO therapeutics is to overcome the biological barriers that prevent them from reaching target sites. This challenge is largely overcome by the backbone, base, and ribose modifications or substitutions as well as utilizing alternative chemistries. While the details of the chemistries and mechanisms are beyond the scope of this review, several reviews can be found that discuss these drug delivery methodologies in depth.^31^, ^32^, ^33^ Extensive studies on the use of nanoparticles^34^ or conjugates^35^ to improve ASO delivery can also be found in the literature.

siRNA therapeutics share similarities to ASOs. siRNAs are double‐stranded RNAs (dsRNAs) that are usually around 20 base pairs long. They are used for gene downregulation or complete silencing. siRNAs comprise of a “passenger” strand and an “antisense” strand that is complementary to the mRNA sequence of interest, similar to ASO‐mediated RNA knockdown or steric translation inhibition (Figure 1). After entering cells, siRNA associates with the Argonaute 2 (AGO2) component of the RNA‐induced silencing complex (RISC).^36^ The passenger strand is then disposed and the antisense strand‐coupled with AGO2 guides the RISC to the mRNA site that is complementary to the antisense strand. This either results in mRNA destruction, in the case of perfect complementarity, or translation inhibition, in the case of imperfect complementarity.^37^ This results in decreased expression of the proteins encoded in the mRNA target. This process is shared by endogenous microRNA (miRNA) and as a result there has been substantial exploration of miRNA mimics.^38^, ^39^ miRNA mimics need to go through an additional modification process by an enzyme called Dicer before being loaded into the RISC.^40^ Many of the previously mentioned ASO modifications or substitutions can be used for siRNA to enhance the stability and protect from endonucleases. For siRNA and miRNA mimics, however, delivery is one of the most significant challenges associated with their clinical translation. Like mRNA, there have been significant explorations into utilizing nanocarriers including liposomes, nanoparticles, and nucleic acid nanostructures, among others.^41^, ^42^ Additionally, bioconjugation to molecules like antibodies, aptamers, peptides, and lipids has also been exploited.^33^, ^43^, ^44^ One of the most successful methods for siRNA delivery thus far has been conjugation with N‐acetylgalactsamine (GalNAc). GalNAc binds the asialoglycoprotein receptor, which is highly expressed in hepatocytes, and allows for efficient uptake of siRNAs bound to it.^45^

The final RNA therapeutics discussed here are aptamers. Aptamers are ssRNAs of 25–80 base pairs that incorporate hairpin folding to form highly specific binding surfaces (Figure 1), analogous to the antigen binding surfaces of antibodies.^46^ They fold into favorable conformations based on complementary base pairing within individual oligo strands. Unlike the previously mentioned RNA therapeutics, aptamers do not need to be delivered into the cell cytosol to take affect and generally have extracellular protein targets.^47^ Aptamers are being used as antagonists to block extracellular interactions, agonists for disease preventing receptors, and as targeted delivery systems for therapeutic molecules such as siRNA, proteins, and small molecule drugs.^46^ While targeted delivery may not be a major challenge for aptamers because of their own high specificity, stability and clearance avoidance in vivo is. Some of the aforementioned nucleobase modifications and substitutions have been proven effective to solving the stability and quick clearance issues. Additionally, conjugation of biocompatible polymers, for example, polyethylene glycol (PEG), to the 5′ end of the aptamer has helped with the clearance issue.^48^

Notably, RNA therapeutics hold several advantages over other therapeutic modalities such as small molecules, antibodies, and DNA therapeutics. One major advantage is their capability to target undruggable targets that conventional therapeutics cannot.^49^ RNA therapeutics, particularly ASOs and siRNAs, interact with their target via sequence‐specific binding. This unique mechanism renders them capable of targeting both noncoding and coding RNAs, which small molecules and antibodies cannot easily achieve.^33^ Because of this, RNA therapeutics are well suited to treat a broad spectrum of diseases including some orphan genetic disorders, which have no other effective therapeutic options. In addition, RNA therapeutics can be modular and versatile in the sense that the RNA sequence and/or delivery system can be easily modified to treat other diseases.^50^ Unlike small molecules and antibodies, which require a long discovery and production process, new RNA therapeutics can be quickly designed and produced using existing modification methods and delivery technologies. This is best exemplified by the rapid development of mRNA‐based COVID‐19 vaccines, which employed new mRNA sequences but existing mRNA modification methods and LNPs for fast clinical testing.^51^ Further, because RNA therapeutics can modulate the protein production/function from the RNA level, they may achieve longer‐lasting effect and reduce administration frequency as compared to conventional therapeutics such as small molecules. This is exemplified by an approved siRNA product, inclisiran, which can maintain its effect for over 6 months following a single‐dose administration.^52^ Moreover, RNA therapeutics usually do not modify the patients' genome and therefore have relatively low risk of genotoxicity as compared to DNA therapeutics and gene editing technologies. Gene editing therapies such as clustered regularly interspaced short palindromic repeats (CRISPR) and RNA editing can provide functions similar to ASO, siRNA, and mRNA using guide DNA/RNA coupled with CRISPR associated proteins (Cas) or adenosine deaminases acting on RNA (ADARs), respectively.^53^ However, CRISPR's permanent genome editing can lead to genotoxicity when off‐target genes are mutated.^54^ Additionally, both CRISPR and RNA editing require more macromolecular machinery to be delivered to the right location inside the target cell than the RNA therapeutics do.^55^ In contrast, RNA therapeutics can offer a safer means to treat genetic disorders.

## 3 APPROVED PRODUCTS

Seventeen RNA therapeutic products using mRNA, ASOs, siRNA, or aptamers have been approved worldwide (Table 2). These therapeutics are used to treat three main disease types: genetic, infectious, and physiological (diseases that cause organ dysfunction but do not fall into the genetic or infectious disease category). Notably, 12 of these approved products were granted orphan designation by the FDA.

### 3.1 Approved mRNA therapeutics

Two mRNA therapeutics (Comirnaty developed by Pfizer‐BioNTech and Spikevax developed by Moderna) have been approved; both are mRNA‐based COVID‐19 vaccines. These vaccines utilize mRNA sequences that code for the SARS‐CoV‐2 spike protein, which is responsible for virus binding to host cells. Upon expression of spike protein analogs by muscle cells and antigen presenting cells local to the intramuscular injection site, patients develop antigen‐specific cellular and humoral immune responses.^56^, ^57^ The important immune cascades involved have been discussed in greater detail elsewhere.^58^, ^59^ Both vaccines code for identical amino acid sequences but differ in antigen‐coding nucleic acid sequences. They also use proprietary 5' UTR and 3' UTR sequences^60^ and lipid nanoparticle delivery vehicles.^61^ LNPs aid in the transfection, endocytosis, and endosomal escape of mRNA in target cells, while the UTR modifications enhance the mRNA translation. Both Comirnaty (tozinameran) and Spikevax were given emergency use authorization by the FDA in December 2020 and granted approval in 2021 and 2022, respectively.

### 3.2 Approved ASO therapeutics

Ten ASO products have been approved worldwide for treating genetic and infectious diseases (Table 1). Nine of these products received orphan status by their respective regulatory agencies. The first RNA therapeutic ever approved and the only ASO approved for infectious diseases was Vitravene (fomivirsen). Vitravene was approved for treating cytomegalovirus (CMV) retinitis in immunocompromised patients. CMV‐infected healthy patients are generally asymptomatic. However, immunocompromised acquired immunodeficiency syndrome (AIDS) patients face more substantial systemic infection with severe inflammation in the eye causing blindness.^62^ Vitravene was developed as an RNA‐knockdown ASO, which targets the mRNA encoding the major immediate‐early region of CMV resulting in decreased viral replication and load. Vitravene is injected intravitreally (IVT) to improve its local targeting to the retina.^63^ However, it was discontinued due to adverse effects at the injection site and liver toxicity.^64^

**TABLE 1:** Clinically approved RNA therapeutics, grouped by RNA therapeutic type

Trade Name (International Nonproprietary Name) *Manufacturer*	Approval Year	Review Priority, Orphan Status	Disease Type	Indication	Administration Route	Delivery System
mRNA						
Comirnaty (tozinameran) *Pfizer‐BioNTech*	2021 (FDA, HC)		Infectious	COVID‐19	Intramuscular	LNP
Spikevax (elasomeran) *Moderna*	2021 (HC); 2022 (FDA)		Infectious	COVID‐19	Intramuscular	LNP
siRNA						
Onpattro (patisiran) *Alnylam*	2018 (EDA, EMA); 2019 (HC, OMDA)	Priority, Orphan	Genetic	Hereditary transthyretin‐mediated amyloidosis	Intravenous	LNP
Givlaari (givosiran) *Alnylam*	2019 (FDA); 2020 (EMA, HC)	Priority, Orphan	Genetic	Acute hepatic porphyria	Subcutaneous	Conjugate (GalNAc)
Oxlumo (lumasiran) *Alnylam*	2020 (FDA, EMA); 2022 (HC)	Priority, Orphan	Genetic	Primary hyperoxaluria type 1	Subcutaneous	Conjugate (GalNAc)
Leqvio (inclisiran) *Novartis*	2020 (EMA); 2021 (FDA, TGA, HC)	Standard	Genetic and Physiological	Heterozygous familial hypercholesterolemia (HeFH) or clinical atherosclerotic cardiovascular disease (ASCVD)	Subcutaneous	Conjugate (GalNAc)
ASO						
Vitravene (fomivirsen) *Isis Pharmaceuticals*‐discontinued	1998 (FDA); 1999 (EMA)	Priority	Infectious	Cytomegalovirus retinitis in immunocompromised patients	Intravitreal	Mod/Sub (PS)
Kynamro (mipomersen) *Genzyme ‐*discontinued	2013 (FDA)	Standard, Orphan	Genetic	Homozygous familial hypercholesterolemia	Subcutaneous	Mod/Subs (2’‐MOE, PS, 5‐methyl cytosine)
Spinraza (nusinersen) *Biogen*	2016 (FDA, EMA); TGA 2017 (TGA, PMDA, HC, Anvisa)	Priority, Orphan	Genetic	Spinal muscular atrophy	Intrathecal	Mod/Subs (2’‐MOE, PS, 5‐methyl cytosine)
Tegsedi (inotersen) *Akcea*	2018 (FDA, EMA); 2019 (HC); 2020 (Anvisa)	Priority, Orphan	Genetic	Hereditary transthyretin‐mediated amyloidosis	Subcutaneous	Mod/Subs (2’‐MOE, PS)
Exondys 51 (eteplirsen) *Sarepta*	2016 (FDA)	Priority, Orphan	Genetic	Duchenne muscular dystrophy (DMD)	Intravenous	Mod/Sus (PMO)
Vyondys 53 (golodisen) *Sarepta*	2019 (FDA)	Priority, Orphan	Genetic	Duchenne muscular dystrophy (DMD)	Intravenous	Mod/Sus (PMO)
Milasen *Brammer Bio*	2018 (FDA); Personalized	Orphan	Genetic	Batten Disease	Intrathecal	Mod/Subs (2'‐MOE, PS, 5‐methyl cytosine)
Viltepso (viltolarsen) NS Pharma	2020 (FDA, PMDA)	Priority, Orphan	Genetic	Duchenne muscular dystrophy (DMD)	Intravenous	Mod/Sus (PMO)
Amondys 45 (casimersen) *Sarepta*	2021 (FDA)	Priority, Orphan	Genetic	Duchenne muscular dystrophy (DMD)	Intravenous	Mod/Sus (PMO)
Waylivra (volanesorsen) *Akcea*	2019 (EMA)	Orphan	Genetic	Familial chylomicronemia syndrome	Subcutaneous	Mod/Subs (2'‐MOE)
Aptamer						
Macugen (pegaptanib) *Gilead* ‐ discontinued	2004 (FDA); 2005 (HC); 2006 (EMA); 2008 (PMDA)	Priority	Physiological	Age‐related macular degeneration	Intravitreal	Conjugate (PEG)

Abbreviations: Regulatory agencies: Anvisa, Brazilian Health Regulatory Agency; EMA, European Medicines Agency; FDA, United States Food and Drug Administration; HC, Health Canada; PMDA, Japanese Pharmaceuticals and Medical Devices Agency. Delivery system: cEt, constrained ethyl bridge nucleic acid substitution; CpG, cytosine phosphodiester guanine; GalNAc, N‐acetylgalactosamine; LNP, lipid nanoparticle; PEG, polyethylene glycol; PMO, phosphordiamidate morpholino alternative chemistry; PS, phosphorothioate backbone modification; 2'‐MOE, 2'‐*O*‐methoxyethyl ribose substitution; 2'‐OMe, 2'‐*O*‐methyl ribose substitutions.

While Vitravene was unsuccessful in the clinic, its landmark approval introduced ASO therapeutics to the clinic. Nine ASO products have since received approvals, all for treating genetic diseases (Table 1). Four of the approved ASOs including Exondys 51 (eteplirsen), Vyondys 53 (golodirsen), Viltepso (viltolarsen), and Amondys 45 (casimersen) were approved for treating Duchenne muscular dystrophy (DMD). DMD is an X‐linked genetic disorder which affects mostly young boys. Muscle weakness is usually noticed at very young ages, with loss of ambulation occurring by age 12, and death between age 20 and 40.^65^ When the gene encoding muscle dystrophin is mutated, the truncated dystrophin prevents proper muscle fiber connection and causes muscular dystrophy.^66^ ASOs used to treat DMD all use splice modulation to exclude the mutated exon of interest resulting in a shortened dystrophin isoform, which can partially or fully restore function.^67^ All four approved ASOs for DMD are phosphordiamidate morpholino oligonucleotides (PMOs). This alternative chemistry replaces the ribose backbone rings with morpholino rings, through a phosphordiamidate linkage, resulting in a more neutral backbone at the physiological pH. This chemistry helps to stabilize the ASOs and protect against proteolytic degradation and nonspecific protein bindinig.^33^, ^68^

Spinraza (nusinersen) is an ASO approved for treating another muscular genetic disorder, spinal muscular atrophy (SMA).^69^, ^70^ SMA stems from genetic mutations or deletions in the gene that encodes the survival motor neuron 1 (SMN1) protein, resulting in loss of function of spinal cord motor neurons. Spinraza uses splice modulation by hybridizing with the SMN2 pre‐mRNA at a location that is a few nucleotides past the 3′ end of exon 7, resulting in occlusion of splice repressors, increased exon 7 inclusion, and production of the SMN1 homolog.^70^ Spinraza utilizes a phosphorothioate (PS) backbone modification, which decreases susceptibility to nucleases, 2'‐O‐methoxyethyl (2'‐MOE) ribose substitutions, which enhance stability and complementary binding, and 5‐methylpyrimidine nucleobase modifications, which increase the ASO melting temperature.^33^, ^71^ Spinraza was approved for intrathecal injection.

Milasen is a personalized medicine approved by the FDA in 2018 for treating Batten's disease in a then 8‐year‐old patient. The patient had an insertion mutation in the gene encoding major facilitator superfamily domain containing 8 (MFSD8) protein, which likely plays a key role in cellular ion transport, that leads to significant central nervous system malfunction.^72^ Milasen utilized the Spinraza scaffold for splice modulation to restore the correct reading frame and translation of MFSD8.^73^ The patient received intrathecal administration every 3 months and experienced significantly fewer seizures with no serious side effects. The patient passed away after 3 years of treatment in 2021. However, this case showed the ability to swiftly (in under a year) develop a personalized ASO treatment for orphan genetic diseases.

Tegsedi (inotersen) is an ASO that was approved for treating transthyretin (TTR)‐mediated familial amyloidotic polyneuropathy (FAP). TTR is an important thyroid hormone transport protein^74^ and mutations in the gene encoding it causes destabilization of the canonical TTR tetramer, resulting in amyloidosis and fibril formation in a variety of vital organs, like the heart, leading to organ failure.^75^ Tegsedi functions by RNA knockdown that binds to mRNA encoding TTR, resulting in decreased levels of TTR and subsequent amyloid formation. Tegsedi uses PS backbone modifications, 2'‐MOE ribose substitutions, and 5‐methylpyrimidine nucleobase modifications, and is administered subcutaneously.^76^

Kynamro (mipomersen) is an ASO that was approved for treating homozygous familial hypercholesterolemia (HoFH). FH results from a mutation in the gene encoding low‐density lipoprotein receptor (LDLR), which decreases its expression. LDLR facilitates LDL cholesterol (LDLC) endocytosis and hepatic clearance. Patients with an under‐expressed LDLR have elevated LDLC levels, which can lead to atherosclerotic cardiovascular disease (ASCVD), from buildup of plaque on blood vessel walls.^77^, ^78^ Homozygous FH (HoFH) is a rarer but more severe form of FH. Kynamro targets the mRNA encoding apolipoprotein B which binds cholesterol to form LDLC and causes RNA knockdown and subsequent downregulation of LDLC. It is injected subcutaneously and incorporates 2'‐OMe ribose substitutions, PS backbone modifications, and 5‐methylpyrimidine nucleobase modifications.^79^ However, it was discontinued in 2016 due to off‐target liver toxicity.^80^

Of the 10 approved ASO products, only one had received approval by the EMA but not the FDA. Waylivra (volanesorsen) is a 2' MOE ribose substituted ASO that was approved for treating familial chylomicronemia syndrome which occurs from loss‐of‐function mutations in the gene encoding lipoprotein lipase, a key enzyme for degradation of triglycerides into free fatty acids. Abnormally high circulating triglyceride can cause pancreatitis.^81^ Waylivra targets the mRNA encoding apolipoprotein C‐III (a component of triglyceride lipoproteins that inhibits its degradation) and uses RNA knockdown to inhibit its translation. This effectively lowers triglyceride levels in circulation after subcutaneous injection.^82^

### 3.3 Approved siRNA therapeutics

Our search identified four approved siRNA products which all received approvals in or after 2018. Onpattro (patisiran) is the first approved siRNA product that was indicated for treating TTR‐FAP. It cleaves mRNA at the complementarity site using the RISC, resulting in decreased TTR expression. Onpattro utilizes 2'‐*O*‐methyl (2'‐OMe) ribose substitutions to enhance stability and complementary base pair binding, is formulated in lipid nanoparticles (LNPs), and is injected intravenously.^83^ Leqvio (inclisiran) is an siRNA that was approved for treating HeFH‐ASCVD and nonhereditary ASCVD. It functions by targeting proprotein convertase subtilisin‐kexin type 9 (PCSK9) transcripts causing RISC‐mediated mRNA degradation. PCSK9 binds LDLR to cause lysosomal degradation of the receptor, thus, knocking down PCSK9 effectively upregulates LDLR.^84^ This allows for increased LDLC endocytosis and decreased serum levels in patients with low LDLR basal levels (HeFH) and elevated LDLC levels (nonhereditary ASCVD).^85^, ^86^ Leqvio is administered subcutaneously and its conjugation to GalNAc allows for efficient targeting to hepatic cells where a majority of LDLR is expressed.

GalNAc conjugate mediated transfection provides the ability to target diseases in hepatic cells. To date, two other siRNA‐GalNAc therapeutics have received approval for modulating hepatic enzyme expression. Givlaari (givosiran) received approval for treating acute hepatic porphyria. Mutation in the delta‐aminolevulinic acid synthase (ALAS) gene causes ALAS overexpression. ALAS is an important enzyme in the heme synthesis pathway and its overexpression can lead to an increase in porphyrins (heme precursors) causing a negative feedback loop.^87^ ALAS1 overexpression and subsequent negative feedback in the liver results in a local heme synthesis dampening and liver heme deficiency. Givlaari targets ALAS1 mRNA leading to decreased translation, no negative feedback, and restoration of normal heme synthesis.^88^ Oxlumo (lumasiran) was approved for treating primary hyperoxaluria type 1 (PH1). PH1 patients have a mutation that decreases alanine‐glyoxylate aminotransferase (AGT) expression causing decreased catalysis of alanine and glyoxylate. The increased glyoxylate can be converted by glycolate oxidase (GO) to oxalate, which can crystalize with calcium and cause organ (commonly kidney) dysfunction with crystal buildup.^89^ Oxlumo downregulates GO expression in the liver by hybridizing with the mRNA encoding hydroxyacid oxidase and results in reduced conversion of glyoxylate to oxalate.^90^ Both Givlaari and Oxlumo are administered subcutaneously for hepatic trafficking.

### 3.4 Approved aptamer therapeutic

Macugen (pegaptanib) is the only aptamer therapeutic approved for treating neovascular macular degeneration (wet AMD). Wet AMD arises from angiogenesis toward the outer retina causing fluid accumulation in and around the retina. This can lead to blurred vision and eventually blindness if left untreated.^91^ Macugen is administered IVT to bind to vascular endothelial growth factor 165 (VEGF165) blocking its interaction with vascular endothelial cells that would normally lead to neovascularization. As a result, it slows or stops the progression of wet AMD.^92^ Macugen has both 2'‐OMe and 2'‐Fluoro ribose modifications for endonuclease protection and is conjugated to PEG to help prevent bulk clearance. However, it was discontinued in 2020 by the manufacturer due to undisclosed reasons.

## 4 CURRENT CLINICAL TRIALS

We performed a search on clinicaltrials.gov to identify active clinical trials on RNA therapeutics. We performed searches in both the “Other Terms” and “Intervention/Treatment” categories, using the terms “RNA therapeutics,” “mRNA,” “siRNA,” “ASO,” “aptamers,” “miRNA,” and alternative versions (e.g., messenger RNA, small interfering RNA, etc.). Under the “Recruitment Status” section we checked “Not yet recruiting,” “recruiting,” “enrolling by invitation,” and “Active, not recruiting.” Also, in the “Study Type” category, we selected “Interventional Studies (Clinical Trials).” We excluded studies that were listed as “Not Applicable” in the “Phase” category. A total of 415 trials were initially identified. We then excluded 43 trials that were regarding cell therapies that incorporated one of the listed RNA therapeutics. Additionally, 189 trials were excluded because they mentioned one of the RNA types but were not regarding treatments, for example, trials that mention measuring endogenous mRNA transcripts. This resulted in 183 trials of interest. We also searched the websites of relevant biotech and pharma companies and found an additional 39 trials, bringing the total to 222 trials. The data were collected in November 2021 and the RNA therapeutic type is summarized in Figure 1.

### 4.1 Overview of current clinical trial landscape for RNA therapeutics

**FIGURE 2:**
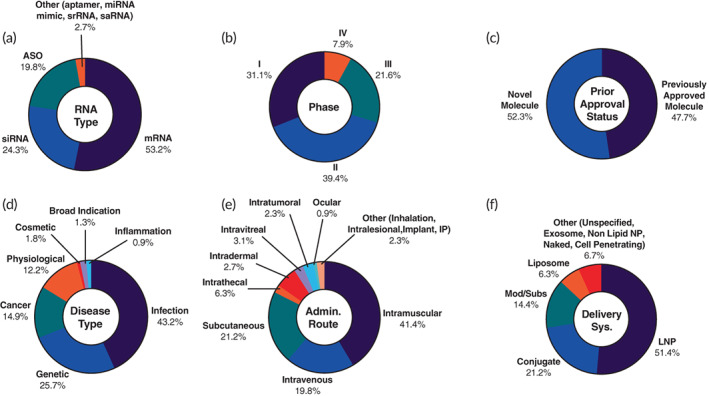
Landscape of RNA therapeutics with 222 ongoing clinical trials. The trials were further analyzed based on (a) RNA type, (b) phase, (c) prior approval status, (d) disease type, (e) administration route, and (f) delivery system

Our analysis showed that the majority of trials (53.2%) are mRNA‐focused with fewer siRNA (24.3%) and ASO (19.8%) trials. Six ongoing trials are outside of these three major RNA therapeutics and related to aptamer, miRNA mimic, self‐replicating RNA (srRNA), and self‐amplifying RNA (saRNA) (Figure 2a, Table 5). Of note is the significant increase in mRNA trials since the emergency use authorization of COVID‐19 vaccines; there were only nine active mRNA trials in 2018.^7^ Impressively, 29.5% of trials are in the late‐stage (Phases 3 and 4) (Figure 2b). A promising sign for RNA therapeutics is that 52.3% of the identified trials are related to new RNA molecules that have not received prior approvals. This means, new therapeutics are still on the horizon and progressing through trials (Figure 2c). We performed an analysis on disease type and found that the largest percentage (43.2%) of trials fell into the infectious disease category, while genetic diseases (25.7%) and cancer (14.9%) make up another significant portion of disease indications (Figure 2d). This is not surprising owing to the ongoing assessment of both new mRNA COVID‐19 vaccines and additional assessment of the Comirnaty and Spikevax in various patient populations, which accounts for 81 trials. With COVID‐19 vaccine trials removed, the three leading indication classes are genetic, cancer, and physiological diseases at 40.4%, 23.4%, and 19.1%, respectively (Figure S1). An additional important clinical parameter is the administration route; intramuscular (IM) injections are the most common (41.4%) in the identified trials (Figure 2e). All IM injections are related to mRNA therapeutics, which is expected to elicit strong immune response for vaccine efficacy. Intravenous (IV) and subcutaneous (SC) injections are used in far fewer trials, 19.8% and 21.2%, respectively (Figure 2e). It is, however, interesting to see the prevalence of SC injection, which can lead to better patient compliance compared to IM or IV.^93^, ^94^ The final characteristic we analyzed was the delivery system (Figure 2f). We and others have discussed the importance of delivery technologies for RNA molecules.^19^, ^20^, ^31^, ^33^, ^34^, ^44^ The clear leaders for RNA delivery in the identified trials are lipid‐based particles, with LNPs accounting for 51.4% and liposomes accounting for 6.3% of trials. Conjugation is also a common delivery method making up 21.2% of the total 222 trials (Figure 2f). However, the relative percentages of delivery technologies, as well as disease indication and administration route, change drastically within each RNA therapeutic subcategory.

### 4.2 Current mRNA clinical trials

**FIGURE 3:**
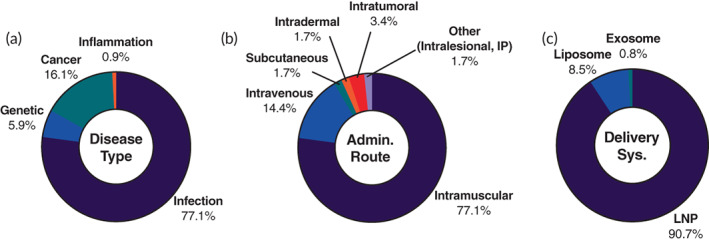
Landscape of 118 ongoing mRNA therapeutic clinical trials. The trials were analyzed based on (a) disease type, (b) administration route, and (c) delivery System

The largest segment of ongoing RNA therapeutic clinical trials is the 118 mRNA‐based trials. A majority of these trials are in early‐phase with 72.5% in Phase 1 or 2. However, the mRNA category has a higher percentage of late‐phase trials than the collective RNA therapeutics (Figure S2a). Of note, 54.2% of the identified active mRNA trials are related to previously approved COVID‐19 vaccines and seeking additional approval for different patient groups (Figure S2b). Additionally, this means that other analysis categories are skewed heavily toward the COVID‐19 vaccine attributes. As a result, the vast majority of mRNA trials have a disease indication of infection, use IM as the administration route, and utilize LNPs as the delivery method (Figure 3a–c). For this reason, we further analyzed the data by eliminating COVID‐19 vaccine trials. This consisted of 66 trials that used either the Spikevax or Comirnaty and 15 trials for new COVID‐19 vaccines. The 66 trials that use either Spikevax or Comirnaty are expanding upon current approvals for use in specific patient populations, for example, patients with hematological malignancies (NCT04847050) or with organ transplants (NCT04885907). Most of the 15 new COVID‐19 vaccine trials are in early‐phase, and only 4 are in Phase 3. While Moderna is the sponsor for 2 of these 15, searching for a more shelf‐stable mRNA vaccine, many of the trials are sponsored by biotech and pharmaceutical companies that do not already have approved products. These companies included Sanofi, CureVac, Arcturus, and others.

**FIGURE 4:**
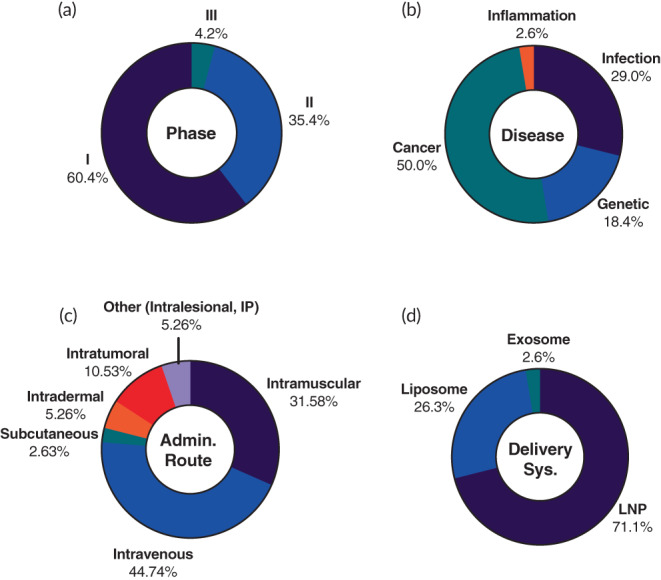
Landscape of 38 ongoing mRNA therapeutic clinical trials that are not for COVID‐19 vaccines. The trials were further analyzed based on (a) phase, (b) disease type, (c) administration route, and (d) delivery system

After removing the COVID‐19 mRNA trials, 38 novel therapeutic trials were left. Most of these trials (95.8%) are in early‐stage with a high percentage (60.4%) of Phase 1 trials (Figure 4a). As expected, there is a substantial change in the disease indication representation. The breakdown consisted of cancers (50%), infectious disease (29%), genetic disorders (18.4%), and 1 inflammatory disease trial (Figure 4b). Cancer indications spanned a range of subtypes with skin cancers (melanoma and squamous cell carcinoma) being the most prominent (36.8%) of the mRNA cancer trials. Melanoma was the indication for the very first mRNA trial in 2008.^95^ Other cancer types included in these trials are glioblastoma, breast cancer, ovarian cancer, prostate cancer, lymphoma, lung cancer, and metastatic cancer (Table 2). Of the mRNA cancer trials, 78.9% are cancer vaccine driven, while the rest are focused on immuno‐oncological treatments. These cancer vaccine trials involve the use of mRNAs encoding tumor‐associated antigens or neoantigens such as human papillomavirus type 16 (HPV‐16) E6 and E7 proteins which are constitutively expressed in HPV‐16 positive cancers (NCT04534205). Immuno‐oncological treatments use mRNAs that encode for immunomodulatory proteins; examples include pro‐inflammatory surface marker OX40L (NCT03323398 and NCT02872025) and pro‐inflammatory cytokine IL‐23 (NCT03739931). Additionally of interest is the fairly even split between the use of liposomes (52.6%) and LNPs (47.4%) as the delivery carriers in the identified mRNA cancer trials.

**TABLE 2:** Selected representative examples of current active mRNA clinical trials

NCT ID (Phase)	Disease type (indication)	Intervention (Sponsor)	Administration Route	Delivery System (Subtype)
NCT04573140 (Phase 1)	Cancer (adult glioblastoma)	mRNA‐loaded DOTAP liposome (CureSearch)	Intravenous	Liposome
NCT02316457 (Phase 1)	Cancer (triple negative breast cancer [TNBC])	IVAC_W_bre1_uID/IVAC_M_uID (BioNTech)	Intravenous	Liposome
NCT02872025 (Early Phase 1)	Cancer (carcinoma, intraductal, noninfiltrating)	mRNA 2752 (Merck Sharp & Dohme Corp., Moderna)	Intralesional	LNP
NCT03897881 (Phase 2)	Cancer (melanoma)	mRNA‐4157 (Moderna, Merck Sharp & Dohme Corp.)	Intravenous	LNP
NCT03871348 (Phase 1)	Cancer (metastatic neoplasm)	SAR441000 (Sanofi, BioNTech)	Intratumoral	LNP
NCT03164772 (Phase 1/2)	Cancer (Metastatic non‐small cell lung cancer)	BI 1361849 (MedImmune, CureVac, PharmaJet)	Intradermal	LNP
NCT03948763 (Phase 1)	Cancer (neoplasms, carcinoma, non‐small‐cell lung cancer, pancreatic neoplasms, colorectal neoplasms)	V941 (Merck Sharp & Dohme Corp.)	Intramuscular	LNP
NCT04163094 (Phase 1)	Cancer (ovarian cancer)	W_ova1 Vaccine (BioNTech)	Intravenous	Liposome
NCT03313778 (Phase 1)	Cancer (solid tumors)	mRNA‐4157 (Moderna, Merck Sharp & Dohme Corp.)	Intramuscular	LNP
NCT04534205 (Phase 2)	Cancer (unresectable head and neck squamous cell carcinoma, metastatic head and neck cancer, recurrent head and neck cancer)	BNT113 (BioNTech)	Intravenous	Liposome
NCT05043181 (Phase 1)	Genetic (familial hypercholesterolemia)	Low‐Density Lipoprotein Receptor mRNA Exosomes (Tang‐Du Hospital, Air Force Military Medical University, China)	Intravenous	Other (Exosome)
NCT04990388 (Phase 1/2)	Genetic (glycogen storage disease type III)	UX053 (Ultragenyx Pharmaceutical)	Intravenous	LNP
NCT04442347 (Phase 1)	Genetic (ornithine transcarbamylase deficiency)	ARCT‐810 (Arcturus Therapeutics)	Intravenous	LNP
NCT05130437 (Phase 1/2)	Genetic (propionic acidemia)	mRNA‐3927 (Moderna)	Intravenous	LNP
NCT04652102 (Phase 2/3)	Infectious (COVID‐19)	CVnCoV (CureVac)	Intramuscular	LNP
NCT05085366 (Phase 3)	Infectious (cytomegalovirus)	mRNA‐1647 (Moderna)	Intramuscular	LNP
NCT05001373 (Phase 1)	Infectiious (HIV)	Core‐g28v2 60mer and eOD‐GT8 60mer (Moderna)	Intramuscular	LNP
NCT04144348 (Phase 1)	Infectious (human metapneumovirus and human parainfluenza)	mRNA‐1653 (Moderna)	Intramuscular	LNP
NCT03713086 (Phase 1)	Infectious (rabies)	CV7202 (CureVac)	Intramuscular	LNP
NCT04528719 (Phase 1)	Infectious (respiratory syncytial virus)	mRNA‐1345 (Moderna)	Intramuscular	LNP
NCT04956575 (Phase 1/2)	Infectious (seasonal influenza)	mRNA‐1010 (Moderna)	Intramuscular	LNP
NCT04917861 (Phase 2)	Infectious (Zika virus)	mRNA‐1893 (Moderna)	Intraperitoneal	LNP
NCT04916431 (Phase 1)	Inflammation (various autoimmune disorders)	mRNA‐6231 (Moderna)	Subcutaneous	LNP

The genetic disorders studied in the identified mRNA trials are mostly endocrine disorders, with one metabolic disorder, ornithine transcarbamylase deficiency (Table 2). One of the trials (NCT04442347) aims to treat FH. This treatment uses exogenous mRNA to restore normal levels of LDLR like Leqvio. The other genetic disease trials utilize mRNA to generate disease‐relevant protein replacement to treat glycogen storage disease type III and various forms of acidemia (Table 2). LNPs are used as the delivery system in all the identified mRNA genetic disease trials except the FH trial (NCT05043181) which uses exosomes as the carrier.

The non‐COVID‐19 infectious disease trials aspire to vaccinate against viruses including cytomegalovirus, rabies, respiratory syncytial virus, zika, influenza and HIV (Table 2). They all have similar mechanisms of action to the approved COVID‐19 mRNA vaccines, delivery of mRNA that codes for some component of the viral capsid of interest to induce an immune response. Interestingly, 7 of the 11 non‐COVID‐19 infectious disease mRNA trials use mRNAs encoding for at least two viral antigens. The use of multiple viral proteins might stimulate multivalent, nonoverlapping immune responses that lead to better viral neutralization effects. For example, one trial (NCT05001373) is studying a vaccine that codes for two HIV glycoproteins for vaccination. The inflammatory disease trial (NCT04916431) utilizes mRNA encoding human interleukin‐2 for upregulation in autoimmune disorders (Table 2).

The most common administration route within the non‐COVID‐19 mRNA trials is IV (44.7%), all for treating diseases of genetic origin and cancer (Figure 4c). Both disease types require systemic protein upregulation for treatment. Specifically, genetic disease targeted mRNA therapeutics act as protein replacement therapeutics, while cancer treatments aim at targeting metastases or other nonresectable tumors. All but two of the trials using IM injections, 31.6% of the non‐COVID‐19 subgroup, are for treating infectious disease (Figure 4c). These numbers are not surprising as IM injections have long been proven an effective method to induce strong immune responses. LNPs (71.1%) are favored as the delivery system in non‐COVID‐19 trials (Figure 4d). Liposomes make up the rest (26.3%) of the identified delivery carriers in this subsection (Figure 4d). Selected ongoing mRNA therapeutic clinical trials can be found in Table 2.

### 4.3 Current ASO clinical trials

**FIGURE 5:**
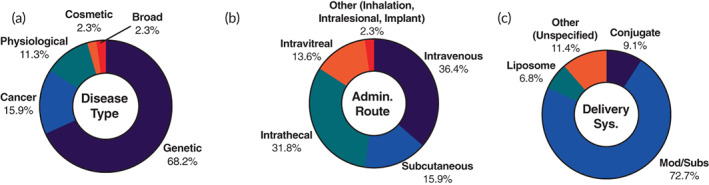
Landscape of 44 ongoing ASO therapeutic clinical trials. The trials were analyzed based on (a) disease type, (b) administration route, and (c) delivery system

A total of 44 active ASO clinical trials were identified. A substantial number (25.0%) of these trials are in late‐phase but are mostly investigating previously approved products for assessing their longer‐term safety/efficacy and their application in different patient populations (Figure S3a). Only three late‐phase trials are related to new ASO molecules. Two late‐phase trials (NCT03913143 and NCT04855045) are investigating new ASOs for treating the ophthalmological genetic disease Leber congenital amaurosis 10 (LCA10) with the third late‐phase trial (NCT05185843) seeking approval for managing familial chylomicronemia syndrome. A fair number of ASO trials (36.4%) are related to previously approved ASO products (Figure S3b) with the purposes of better assessing long‐term efficacy and safety. Unlike the approved products, which are exclusively used to treat genetic disorders, only 68.2% of the identified active ASO trials are for genetic disease treatment (Figure 5a). The genetic diseases investigated for the new ASOs are mostly ophthalmological and neurological disorders. Retinitis pigmentosa, which affects the retinal pigment epithelium and can cause blindness, and LCA10, which affects transduction and transport within cone cells of the eye, are the most common ophthalmological diseases with ASO treatments in the clinic (Table 3).^96^, ^97^ Additionally, all the ophthalmological genetic treatments are injected IVT. Neurological diseases of particular interest in the identified ASO trials are Angelman syndrome and DMD (Table 3). Angelman syndrome is characterized by a mutation in the maternal ubiquitin‐protein ligase 3A gene (UBE3A), limiting its expression and synapse formation.^98^ These ASOs function by silencing the paternal version of this gene to allow for upregulation of the UBE3A protein.^99^

**TABLE 3:** Selected presentative examples of current active ASO clinical trials

NCT ID (Phase)	Disease type (indication)	Intervention (Sponsor)	Administration	Delivery System (Detailed)
NCT05018533 (Phase 1)	Broad indication (healthy volunteer)	TAKC‐02 (TAK‐Circulator Co.)	Inhalation	Other
NCT02781883 (Phase 2)	Cancer (acute myeloid leukemia)	BP1001 (Bio‐Path Holdings)	Intravenous	Liposome
NCT04504669 (Phase 1)	Cancer (clear cell renal cell cancer, non‐small‐cell lung cancer, triple negative breast neoplasms, squamous cell cancer of head and neck, small cell lung cancer, gastroesophageal cancer, melanoma, cervical cancer, advanced solid tumors)	AZD8701 (AstraZeneca)	Intravenous	Mod/Subs (cEt)
NCT04072458 (Phase 1)	Cancer (mantle cell lymphoma, peripheral T‐cell lymphoma [PTCL], cutaneous T‐cell lymphoma [CTCL], chronic lymphocytic leukemia (CLL), small lymphocytic lymphoma (SLL), follicular lymphoma, marginal zone lymphoma, Hodgkin lymphoma, waldenstrom macroglobulinemia, diffuse large B‐cell lymphoma)	L‐Bcl‐2 antisense oligonucleotide (Bio‐Path Holdings)	Intravenous	Liposome
NCT04740476 (Phase 2)	Genetic (Dravet syndrome)	STK‐001 (Stoke Therapeutics)	Intrathecal	Mod/Subs (PS‐2'‐MOE)
NCT05032196 (Phase 1/2)	Genetic (Huntington disease)	WVE‐003 (Wave Life Sciences)	Intrathecal	Mod/Subs (PS)
NCT03780257 (Phase 1/2)	Genetic (Retinitis pigmentosa, usher syndrome type 2, deaf blind, retinal disease, eye diseases, vision disorders)	QR‐421a (ProQR Therapeutics)	Intravitreal	Mod/Subs (PS‐2'‐OMe)
NCT03913143 (Phase 2/3)	Genetic (Leber congenital amaurosis 10)	Sepofarsen (ProQR Therapeutics)	Intravitreal	Mod/Subs (PS‐2'‐OMe)
NCT04906460 (Phase 1/2)	Genetic (Duchenne muscular dystrophy)	WVE‐N531 (Wave Life Sciences)	Intravenous	Mod/Subs (PS)
NCT04931862 (Phase 1/2)	Physiological (amyotrophic lateral sclerosis, frontotemporal dementia)	WVE‐004 (Wave Life Sciences)	Intrathecal	Mod/Subs (PS)
NCT03702829 (Phase 2)	Physiological (amyloidosis)	Inotersen (Brigham and Women's Hospital)	Subcutaneous	Mod/Subs (2'‐MOE)
NCT04539041 (Phase 1)	Physiological (progressive supranuclear palsy)	Unnamed ASO (Novartis)	Intrathecal	Other (unspecified)

Furthermore, cancer (15.9%) and physiological diseases (11.3%) make up most of the rest of the ASO trials (Figure 5a). The cancer trials utilize RNA‐knockdown ASOs targeted at oncogenes like B‐cell lymphoma 2 (NCT04072458) and genes associated with cancer cell growth and proliferation mediators like growth factor receptor‐bound protein 2 (NCT02781883 and NCT04196257) and androgen receptor (NCT03300505). All the identified ASO cancer trials use IV as the administration method and are mostly aimed at treating metastatic neoplasms with one trial each for treating prostate cancer (NCT03300505), leukemia (NCT02781883), and lymphoma (NCT04072458) (Table 3). The physiological disease‐treating ASOs include trials for liver‐targeted lipid down‐regulators, and intrathecally injected treatments for Amyotrophic Lateral Sclerosis (ALS) and Progressive Supranuclear Palsy (Table 3). There is only one trial (NCT05018533) with a broad indication.

The administration method for these ASO trials is mostly IV (36.4%), intrathecal (31.8%), IVT (13.6%), or SC (15.9%) (Figure 5b). All of the administration routes correlated well with the desired targeting: IV for systemic delivery, intrathecal for neurological indications, IVT for ophthalmological indications, and SC for liver trafficking and hepatic uptake. Only one (NCT05018533) of the identified ASO trials use inhalation administration for targeted delivery to the lungs. This Phase 1 trial is for broad indications but will aim to treat severe asthma in later phases. It utilizes an ASO that limits MEX3B, an RNA binding protein, whose inhibition could have extensive applications in oncology and infectious disease.^100^ Modifications and substitutions with no carrier (72.7%) are still heavily favored as delivery systems for ASOs (Figure 5c). A common modification in these ASO trials that we have not previously mentioned is the constrained ethyl bridge nucleic acid substitution (cEt), which provides nuclease protection and enhances complementary binding.^6^ We broke down the substitution and modification methods even further (Figure S4). Of the ASO trials that utilized modifications or substitutions, 65.6% had a PS backbone modification, 56.3% had a 2′ ribose substitution (either 2'‐MOE or 2'‐OMe), and 15.6% had a cEt bridged nucleic acid substitution. Liposomes (6.8%) and conjugates (9.1%) are also being explored in the clinic as ASO delivery carriers (Figure 5c). Selected ongoing ASO therapeutic clinical trials can be found in Table 3.

### 4.4 Current siRNA clinical trials

Accounting for ~25% of the ongoing RNA therapeutic trials are 54 siRNA trials. These trials are mostly comprised of Phase 1 (25.5%), Phase 2 (32.7%), and Phase 3 (40.0%) (Figure S5a) with only one Phase 4 trial. Most of the Phase 3 trials, 18 of 22, are related to previously approved products seeking additional approval for either other indications or different treatment regimens. However, 53.7% of the trials are for novel siRNA therapeutics (Figure S5b). The previously approved siRNAs with ongoing trials include Leqvio, Oxlumo, Onpattro, and Givlaari. The Leqvio trials are seeking approval for HoFH and other non‐FH‐derived cardiovascular diseases. The Oxlumo studies are largely focused on better characterizing its PK, PD, and efficacy but also include a new indication for treating patients with high risk of kidney stone formation (NCT05161936). The Onpattro trials focus on assessing its long‐term effect, studying its treatment outcomes in patients with liver transplant, and investigating its application for a new indication (nonhereditary TTR‐FAP). The single Givlaari study (NCT02949830) is to assess its long‐term safety and adverse events.

**FIGURE 6:**
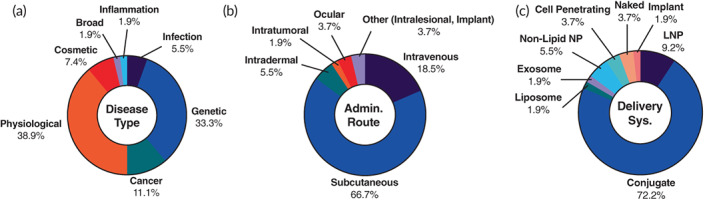
Landscape of 54 ongoing siRNA therapeutic clinical trials. The trials were analyzed based on (a) disease type, (b) administration route, and (c) delivery system

Genetic diseases (33.3%) and physiological diseases (38.9%) are the clear leaders in disease categories (Figure 6a). All of the genetic diseases that these siRNA trials are attempting to treat including FH, PH1, and TTR‐FAP (Table 4) have been discussed in the previous section. The physiological diseases investigated in the identified siRNA trials are comprised of mostly cardiovascular diseases and hepatic diseases (Table 4). The siRNA cardiovascular disease trials explore the use of anti‐hypertensive molecules and treatments for nonhereditary hypercholesterolemia. Hepatic diseases explored in these clinical trials are nonalcoholic fatty liver disease (NAFLD) and non‐alcoholic steatohepatitis (NASH). Both NAFLD and NASH are active areas of research at biotechnology and pharmaceutical companies because it is a fast‐approaching epidemic in the United States.^101^, ^102^ These NAFLD/NASH trials use siRNA to block collagen expression in local hepatic cells.^103^

**TABLE 4:** Selected representative examples of current active siRNA clinical trials

NCT ID (Phase)	Disease type (indication)	Intervention (Sponsor)	Administration	Delivery System (Detailed)
NCT01591356 (Phase 1)	Cancer (advanced malignant solid neoplasm)	EphA2‐targeting DOPC‐encapsulated siRNA (National Cancer Institute)	Intravenous	Liposome
NCT03819387 (Phase 1)	Cancer (non‐small cell lung cancer, pancreatic cancer, colorectal cancer)	NBF‐006 (Nitto BioPharma)	Intravenous	LNP
NCT01676259 (Phase 2)	Cancer (pancreatic ductal adenocarcinoma, pancreatic cancer)	siG12D‐LODER (Silenseed Ltd)	Implant	Other
NCT04995536 (Phase 1)	Cancer (recurrent B‐cell non‐Hodgkin lymphoma)	CpG‐STAT3 siRNA (National Cancer Institute)	Intratumoral	Conjugate (CpG oligodeoxynucleotide)
NCT04844840 (Phase 2)	Cosmetic (Keloid)	STP705 (Sirnaomics)	Intradermal	Other (Non Lipid NP)
NCT03814187 (Phase 3)	Genetic (atherosclerotic cardiovascular disease, elevated cholesterol, heterozygous familial hypercholesterolemia, homozygous familial hypercholesterolemia)	Inclisiran (Novartis)	Subcutaneous	Conjugate (GalNAc)
NCT04666298 (Phase 2)	Genetic (hypercholesterolemia, heterozygous familial hypercholesterolemia)	Inclisiran (Novartis)	Subcutaneous	Conjugate (GalNAc)
NCT03759379 (Phase 3)	Genetic (amyloidosis, hereditary, transthyretin amyloidosis)	Patisiran and Vutrisiran (Alnylam Pharmaceuticals)	Intravenous	Conjugate (GalNAc)
NCT04042402 (Phase 3)	Genetic (primary hyperoxaluria type 1, primary hyperoxaluria type 2, kidney diseases, urologic diseases, genetic disease)	DCR‐PHXC (Dicerna Pharmaceuticals)	Subcutaneous	Conjugate (GalNAc)
NCT04153149 (Phase 3)	Genetic (transthyretin amyloidosis [ATTR] with cardiomyopathy)	Vutrisiran (Alnylam Pharmaceuticals)	Subcutaneous	Conjugate (GalNAc)
NCT02949830 (Phase 1/2)	Genetic (acute intermittent porphyria)	Givosiran (Alnylam Pharmaceuticals)	Subcutaneous	Conjugate (GalNAc)
NCT03681184 (Phase 3)	Genetic (primary hyperoxaluria type 1)	Lumasiran (Alnylam Pharmaceuticals)	Subcutaneous	Conjugate (GalNAc)
NCT03772249 (Phase 1)	Infectious (hepatitis B, chronic)	DCR‐HBVS (Dicerna Pharmaceuticals)	Subcutaneous	Conjugate (GalNAc)
NCT04819269 (Phase 3)	Inflammation (dry eye disease, Sjogren syndrome)	Tivanisiran (Sylentis)	Ocular	Other (Naked)
NCT04601844 (Phase 1)	Broad indications	Cemdisiran (Alnylam Pharmaceuticals)	Subcutaneous	Conjugate (GalNAc)
NCT03705234 (Phase 3)	Physiological (atherosclerotic cardiovascular disease)	Inclisiran (Novartis)	Subcutaneous	Conjugate (GalNAc)
NCT04267393 (Phase 2)	Physiological (nonalcoholic steatohepatitis [NASH])	BMS‐986263 (Bristol‐Myers Squibb)	Intravenous	LNP
NCT03841448 (Phase 2)	Physiological (IgA nephropathy [IgAN], Berger disease, glomerulonephritis)	Cemdisiran (Alnylam Pharmaceuticals)	Subcutaneous	Conjugate (GalNAc)

Additionally, 11.1% of the identified siRNA trials focus on cancer (Figure 6a). All of these trials used siRNA to target and inhibit expression of proteins, whose overexpression are associated with some cancer type. Examples of these protein targets include tyrosine kinase, glutathione S‐transferase P, transforming growth factor beta 1, and others. In the trials we identified, four trials are seeking approval for esthetic use, in which different siRNA molecules are studied for scar removal by local intradermal or SC administration. Subcutaneous injections (66.7%) prevailed as the administration method in the siRNA trials (Figure 6b). Apart from one trial (NCT04707131), an aforementioned local scar‐preventing therapy, all of the siRNA trials using SC administration are related to GalNAc conjugates. Since most of the diseases that these SC siRNAs trials are attempting to treat are of hepatic origin, liver targeting is required. This is easily achieved by SC injections and the siRNA (around 14 kDa) can traffic from the subcutaneous space through the blood capillary tight junctions, which have a molecular weight cutoff of around 20 kDa, into the blood stream to reach the liver.^6^, ^104^ A substantial cohort (18.5%) of the siRNA trials are administered through IV injection for a variety of cancers and hepatic diseases (Figure 6b). Consistent with the approved siRNA delivery vehicles, 72.2% of the trials use siRNA conjugates (Figure 6c). All but one of those are GalNAc conjugates (Figure S6). Additional delivery systems in the siRNA trials include LNPs (9.2%), liposomes (1.9%), or others (16.7%) (Figure 6c). The other delivery vehicles used include nonlipid nanoparticles, naked siRNA, cell penetrating moieties, and liposome‐like exosomes.^105^ Selected ongoing siRNA clinical trials can be found in Table 4.

### 4.5 Other clinical trials

Six of the identified RNA therapeutic trials involve RNA types beyond mRNA, ASOs, and siRNA (Table 5). All but one trial (NCT02321267) are in early‐phase and related to novel therapeutics. The only Phase 4 trial (NCT02321267) focuses on comparing Macugen (an approved aptamer product) to other clinical standards for macular diseases. The two other aptamer trials follow suit of their Macugen predecessor and are both PEG conjugates, which are investigated for treating hereditary clotting disorders (NCT04677803) or targeting the complement component C5a for broad indications (NCT05018403). The single miRNA mimic trial (NCT04675996) is focused on treating bone metastases, in which the mechanism of action of the miRNA mimic is to induce apoptosis and cell‐cycle disruption in cancer cells for sufficient T‐cell response. The last two trials in this category utilize srRNA (NCT04863131) and saRNA (NCT04934111) for COVID‐19 vaccines. Functioning similar to mRNA, srRNA and saRNA can self‐replicate to increase antigen expression and thus elicit better immune responses.

**TABLE 5:** List of current active RNA therapeutic clinical trials that are not for mRNA, ASO, or siRNA

RNA type	NCT ID (Phase)	Disease type (indication)	Intervention (Sponsor)	Administration	Delivery system (Detailed)
aptamer	NCT02321267 (Phase 4)	Physiological (Macular Diseases)	Pegaptanib (Kagawa University)	Intravitreal	Conjugate (PEG)
aptamer	NCT04677803 (Phase 2)	Genetic (Von Willebrand diseases, hemophilia A)	BT200 (Medical University of Vienna)	Subcutaneous	Conjugate (PEG)
aptamer	NCT05018403 (Phase 1)	Other (Healthy)	AON‐D21 (Aptarion Biotech)	Intravenous	Conjugate (PEG)
miRNA mimic	NCT04675996 (Phase 1)	Cancer (Solid tumor)	INT‐1B3 (InteRNA)	Intravenous	LNP
srRNA	NCT04863131 (Phase 1/2)	Infectious (COVID‐19)	EXG‐5003 (Elixirgen Therapeutics)	Intradermal	Other
saRNA	NCT04934111 (Phase 1)	Infectious (COVID‐19)	LNP‐nCOV saRNA‐02 Vaccine (LSHTM Uganda Research Unit)	Intramuscular	LNP

## 5 TRANSLATIONAL CHALLENGES

While the clinical trials and approvals demonstrate the promise of RNA therapeutics, their development is still relatively in the early‐stage when compared to small molecule drugs or protein biologics, which each had 34 and 14 new approvals, respectively, in 2021 alone.^106^ This is in part due to the significant number of translational challenges that still lay ahead. While synthesis of RNA therapeutics may be easier than that of therapeutic proteins like monoclonal antibodies, the purification process is not straightforward. RNA therapeutics can be synthesized at large scale in batches using in vitro transcription (IVTrnsc). IVTrnsc is a cell‐free process, which utilizes specific RNA polymerases, nucleotide triphosphate substrates, a DNA template, and buffer components to create RNA molecules.^107^ IVTrnsc is a much easier process than therapeutic protein synthesis, which usually relies on immortalized cell lines to be closely maintained over a number of weeks. However, at the end of IVTrnsc, the RNA therapeutic must not only be separated from the unreacted synthesis components but many of the RNA therapeutic molecules discussed need to be either encapsulated or conjugated. Purification is often done with chromatographic methods. Ion exchange and reverse‐phase chromatography can be used to remove impurities from large volumes of drug product at a relatively high throughput but cannot remove most stubborn impurities. Affinity chromatography, on the other hand, produces highly purified RNA, but has a low throughput slowing down large‐scale manufacturing.^107^ A major step that the industry can take to alleviate the purification challenges is to implement continuous manufacturing practices, which have been shown to make a significant impact on therapeutic protein production.^108^ While the additional step of LNP encapsulation with microfluidic devices, which has been performed at the largest of scales with Comirnaty and Spikevax, and conjugation, which is a relatively simple process in the context of GalNAc, may not be a bottle neck in terms of production time, it does provide another opportunity for nucleases to cleave RNA therapeutics. Keeping this and other RNA degrading enzymes out of the manufacturing process is one of the most important components of the RNA therapeutic manufacturing process.^109^ Proper assessment of nuclease levels at every step of manufacturing can help mitigate the exposure to degrading enzymes.

Additionally, formulation for enhanced stability and delivery is certainly a challenge for RNA therapeutics to advance in clinical trials and to treat broader diseases. While GalNAc conjugates are widely used for siRNA, these only provide targeted delivery to the liver. Many of the ongoing trials use an administration route for improving delivery to the desired tissue, for example, all ophthalmological RNA therapeutics are administered IVT or intraocularly. In addition, vehicles may need to be designed based on the target cell of interest. For example, LNPs designed to transfect antigen‐presenting cells for vaccine may not work well when the goal is to produce cytokines like interleukin 2, mostly expressed by T cells.^110^ While extensive ongoing research aims to design tissue‐ and cell‐specific targeting systems,^50^, ^111^, ^112^, ^113^ this challenge is exacerbated by the progression from in vitro to preclinical to clinical studies. In vitro studies, like cell culture transfection studies, often do not recapitulate the actual in vivo transfection efficacy. This problem of recapitulation also extends to safety, off‐target immunogenicity, and toxicity.^114^ While animal models can provide significant insight into the safety of a drug of interest, they can hardly tell the full story. Since all RNA therapeutics highlighted in this review modulate protein function and translation, any off‐target effects can lead to localized signaling imbalances in non‐disease‐causing organs. As discussed in Section 3, two approved ASO products (Vitravene and Kynamro) were discontinued due to hepatic toxicity.^64^, ^80^ Additionally, there have been a number of reports that risk of myocarditis is increased with Comirnaty and Spikevax vaccination,^115^, ^116^, ^117^ but additional retrospective analysis with larger sample cohorts and less voluntary reporting should be performed to quantify the true added risk. One mitigation strategy for off‐target toxicity is highly selective targeted delivery, which GalNAc‐conjugates seem to have achieved for hepatic‐associated diseases. But the approved GalNAc therapies, Givlaari, Oxlumo, and Leqvio, have only been on the market for a couple of years, so time will tell whether long‐term dosing is safe. Better models of human and interorgan biology can help to bridge the gap in safety assessments. Researchers have long been working toward organ‐on‐a‐chip and eventually body‐on‐a‐chip in vitro models that can aid in understanding the impacts of new medcines,^118^ if successful this will prove critical to elucidating the safety and efficacy of RNA therapeutics.

Additional challenges that RNA therapeutics face is the storage and stability issue. Generally, cold chain conditions are used to mitigate degradation and aggregation of RNA therapeutic molecules and their carriers.^119^ Currently, for long‐term storage, Comirnaty and Spikevax must be kept at −90 to −60°C and −50 to −15°C, respectively. These are temperature requirements that some medical facilities, especially ones in rural areas or developing countries, do not have the means to adhere to. Comirnaty and Spikevax can be stored in a refrigerator (2–8°C) for 30 days after thawing but must be used within 30 min and 24 h, respectively, after removal from cold chain. In contrast, all the approved ASOs and siRNAs are stored in the refrigerator until vial expiry, with some capable of being stored at room temperature (below 25°C) for up to 6 weeks (Tegsedi and Waylivra) or longer (Leqvio). However, the storage‐stability profiles for ASOs and siRNAs could change with the approval of different drug carriers. Moderna's next generation refrigerator‐stable (2–5°C) COVID‐19 vaccine (mRNA‐1283), which is in Phase 2 trials (NCT04813796 and NCT05137236), could be pivotal for mRNA use in communities where refrigerator storage is more accessible than freezer storage. However, with research in the past largely focusing on naked mRNA storage,^120^ additional efforts need to be made to push the stability and storage boundaries of encapsulated RNA therapeutics.

## 6 CONCLUSION AND OUTLOOK

Despite all the challenges, RNA therapeutics are emerging as a major therapeutic modality. The ability to control protein expression has broad reaching impacts in diverse diseases, as highlighted by the wide range of diseases discussed in this review. With treatment modalities now approved in each of the major RNA therapeutic categories and significant academic and industrial research into improving their clinical use, RNA therapeutics are poised to revolutionize the way diseases can be treated. While the 1990s and 2000s introduced RNA therapeutics to the clinic, the 2010s provided only a few approvals, mostly in orphan genetic diseases, the early progress since 2020 and clinical trials highlighted here show that the next decade should prove monumental in the evolution of RNA therapeutics. While this process will be met with challenges in manufacturing, delivery, and safety, it will be an exciting process to see unfold in preclinical and clinical trials.

## AUTHOR CONTRIBUTIONS

**Alexander Curreri:** Data curation (lead); formal analysis (lead); methodology (equal); validation (equal); writing – original draft (lead); writing – review and editing (equal). **Disha Sankholkar:** Data curation (supporting); formal analysis (equal); methodology (equal); writing – original draft (supporting). **Samir Mitragotri:** Conceptualization (equal); supervision (equal); writing – review and editing (equal). **Zongmin Zhao:** Conceptualization (equal); supervision (equal); validation (equal); writing – original draft (supporting); writing – review and editing (equal).

### PEER REVIEW

The peer review history for this article is available at https://publons.com/publon/10.1002/btm2.10374.

## Supporting information


**Figure. S1** Landscape of disease indications for 141 ongoing clinical trials for RNA therapeutics that are not COVID‐19 vaccines.
**Figure S2.** Landscape of mRNA therapeutics with 118 ongoing clinical trials. The trials were analyzed based on (**a**) Phase and (**b**) Prior Approval Status.
**Figure S3.** Landscape of ASO therapeutics with 44 ongoing clinical trials. The trials were analyzed based on **(a**) Phase and (**b**) Prior Approval Status.
**Figure S4.** Breakdown of the Modification and Substitution types utilized in ongoing ASO clinical trials. (**a**) Backbone modifications. (**b**) Ribose substitutions. (**c**) Bridge substitutions.
**Figure S5.** Landscape of siRNA therapeutics with 54 ongoing clinical trials. The trials were analyzed based on (**a**) Phase and (**b**) Prior Approval Status.
**Figure. S6.** Breakdown of the conjugate types utilized in ongoing siRNA clinical trials.

## Data Availability

Data sharing is not applicable to this article as no new data were created or analyzed.
