# Supplementary material for: RNA therapeutics in the clinic
Source: Bioeng Transl Med. 2022 Jul 6;8(1):e10374. doi: 10.1002/btm2.10374 (PMC9842029; doi:10.1002/btm2.10374)
Supplement: Supplementary file 1 — Figure. S1 Landscape of disease indications for 141 ongoing clinical trials for RNA therapeutics that are not COVID‐19 vaccines. Figure S2. Landscape of mRNA therapeutics with 118 ongoing clinical trials. The trials were analyzed based on (a) Phase and (b) Prior Approval Status. Figure S3. Landscape of ASO therapeutics with 44 ongoing clinical trials. The trials were analyzed based on (a) Phase and (b) Prior Approval Status. Figure S4. Breakdown of the Modification and Substitution types utilized in ongoing ASO clinical trials. (a) Backbone modifications. (b) Ribose substitutions. (c) Bridge substitutions. Figure S5. Landscape of siRNA therapeutics with 54 ongoing clinical trials. The trials were analyzed based on (a) Phase and (b) Prior Approval Status. Figure. S6. Breakdown of the conjugate types utilized in ongoing siRNA clinical trials. [file BTM2-8-e10374-s001.docx]

***Supplementary Information***

**RNA Therapeutics in the Clinic**

Alexander Curreri^1,2^, Disha Sankholkar^3^, Samir Mitragotri^1,2,*^, Zongmin Zhao^4,5,*^

1. John A. Paulson School of Engineering and Applied Sciences, Harvard University, Cambridge, MA 02138

2. Wyss Institute for Biologically Inspired Engineering at Harvard University, Boston, MA 02115

3. Lexington High School, Lexington, MA 02421

4. Department of Pharmaceutical Sciences, College of Pharmacy, University of Illinois at Chicago, Chicago, IL 60612

5. University of Illinois Cancer Center, Chicago, IL 60612

*Correspondence: [mitragotri@seas.harvard.edu](mailto:mitragotri@seas.harvard.edu) (Samir Mitragotri); [zhaozm@uic.edu](mailto:zhaozm@uic.edu) (Zongmin Zhao)

**Figure. S1.** Landscape of disease indications for 141 ongoing clinical trials for RNA therapeutics that are not COVID-19 vaccines.

**Figure S2.** Landscape of mRNA therapeutics with 118 ongoing clinical trials. The trials were analyzed based on **a**) Phase and **b**) Prior Approval Status.

**Figure S3.** Landscape of ASO therapeutics with 44 ongoing clinical trials. The trials were analyzed based on **a**) Phase and **b**) Prior Approval Status.

**Figure S4.** Breakdown of the Modification and Substitution types utilized in ongoing ASO clinical trials. **a**) Backbone modifications. **b**) Ribose substitutions. **c**) Bridge substitutions.

**Figure S5.** Landscape of siRNA therapeutics with 54 ongoing clinical trials. The trials were analyzed based on **a**) Phase and **b**) Prior Approval Status.

**Figure. S6.** Breakdown of the conjugate types utilized in ongoing siRNA clinical trials.
